# Supplementary material for: Induction of Broadly Cross-Reactive Antibodies by Displaying Receptor Binding Domains of SARS-CoV-2 on Virus-like Particles
Source: Vaccines (Basel). 2022 Feb 16;10(2):307. doi: 10.3390/vaccines10020307 (PMC8876827; doi:10.3390/vaccines10020307)
Supplement: Supplementary file 1 [file vaccines-10-00307-s001.zip › vaccines-1551154-supplementary.pdf]

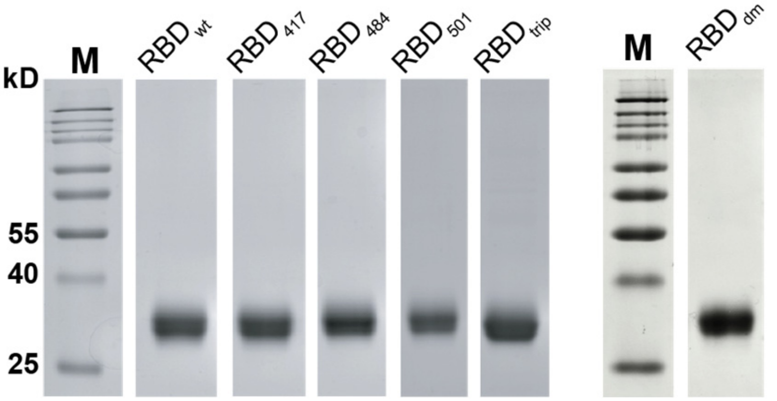

Figure S1. SDS-PAGE gel of RBD_{wt}, RBD_{417}, RBD_{484}, RBD_{501}, RBD_{trip} and RBD_{dm}
